# Evaluation of immunohistochemistry using two different antibodies and procedures for primary lung adenocarcinoma harboring anaplastic lymphoma kinase rearrangement

**DOI:** 10.3892/ol.2014.2511

**Published:** 2014-09-08

**Authors:** JUN AKIBA, AKIHIKO KAWAHARA, HIDEYUKI ABE, KOICHI AZUMA, TOMOHIKO YAMAGUCHI, TOMOKI TAIRA, CHIHIRO FUKUMITSU, YORIHIKO TAKASE, MAKIKO YASUMOTO, YUMI UMENO, KEITA TODOROKI, TAKASHI KURITA, RIN YAMAGUCHI, MASAYOSHI KAGE, HIROHISA YANO

**Affiliations:** 1 Department of Pathology, Division of Respirology, Neurology, and Rheumatology, Kurume University School of Medicine, Kurume, Fukuoka 830-0011, Japan; 2 Department of Diagnostic Pathology, Kurume University Hospital, Kurume, Fukuoka 830-0011, Japan; 3 Department of Internal Medicine, Division of Respirology, Neurology, and Rheumatology, Kurume University School of Medicine, Kurume, Fukuoka 830-0011, Japan

**Keywords:** lung cancer, adenocarcinoma, anaplastic lymphoma kinase, fluorescence *in situ* hybridization, immunohistochemistry

## Abstract

Rearrangements of anaplastic lymphoma kinase (ALK) have been recently identified in non-small cell lung carcinomas. Previous studies have revealed characteristic features, including adenocarcinoma histology and mucin production, in ALK-positive lung carcinoma. The present study evaluated immunohistochemistry (IHC) in ALK-positive lung carcinoma using two different antibodies, clone 5A4 and D5F3, and compared the results. On the basis of the aforementioned characteristic features, out of 359 primary lung carcinomas, the ALK status of 14 adenocarcinomas was screened using the intercalated antibody-enhanced polymer (iAEP) method with antibody 5A4, and this was compared with the ALK status obtained using rabbit monoclonal antibody D5F3 and fluorescence *in situ* hybridization for ALK. Eight cases were demonstrated to be ALK-positive by IHC. Seven cases exhibited ALK rearrangement, which was demonstrated by fluorescence *in situ* hybridization. The IHC for ALK obtained using D5F3 was comparable with that of the iAEP and exhibited low heterogeneity. This finding suggests that IHC for ALK could be useful in limited tissue samples, such as biopsy specimens or cytology, for the screening of ALK-positive lung carcinoma. In the present study, it was demonstrated that IHC with ALK monoclonal antibody D5F3 was useful for screening lung adenocarcinoma harboring ALK rearrangement.

## Introduction

Lung cancer is the most common cause of cancer-associated mortality worldwide (1). Non-small cell lung carcinoma (NSCLC) is a major type of lung cancer. Out of all the NSCLCs, adenocarcinoma is the most common histological type (2). The introduction of the epidermal growth factor receptor (EGFR) tyrosine kinase inhibitors (TKIs) and the approval of their clinical use has provided novel insights into the treatment of advanced NSCLC (3,4). EGFR mutation is a validated predictive marker for response and progression-free survival when using EGFR-TKIs during first-line therapy in advanced lung adenocarcinoma (4–6).

Soda *et al* reported that a minority of lung tumors harbored a small inversion within chromosome 2p, giving rise to echinoderm microtubule-associated protein-like 4 (EML4)-anaplastic lymphoma kinase (ALK), a transformation fusion gene (7). The epidemiological characteristics exhibit prevalence in 5% of adenocarcinomas. The presence of the EML4-ALK fusion is associated with younger, male patients who have no smoking history or a light smoking habit (8–11). Common features of lung carcinoma harboring the ALK-fusion gene include the absence of lepidic growth and marked nuclear pleomorphism, a solid or acinar growth pattern, a substantial amount of extracellular mucus and the presence of mucus cells (12). In addition, a solid signet-ring cell pattern and a mucinous cribriform pattern are observed at least focally in the majority of cases. Tumors with EML4-ALK translocations appear to be exclusive of EGFR and KRAS mutations (8,11,13). The first ALK inhibitor to be used in a clinical trial was crizotinib, which is a dual inhibitor for ALK and MET kinase (14). The response rate for crizotinib in patients with ALK-rearranged NSCLCs in the trial was revealed to be 57%, with a disease control rate of up to 90% (10). Therefore, it is necessary to develop a feasible method of detecting ALK rearrangement.

In the present study, cases harboring ALK rearrangement were selected on the basis of previously documented characteristic features, including adenocarcinoma histology and mucin production. Using this cohort, the correlation between two different immunohistochemistry (IHC) procedures was examined, including the intercalated antibody-enhanced polymer (iAEP) method with antibody 5A4 (Nichirei Biosciences, Inc., Tokyo, Japan) and the fully automated Bond-Max system (Leica Biosystems Newcastle, Ltd., Newcastle Upon Tyne, UK) with rabbit monoclonal antibody D5F3 (Cell Signaling Technology, Inc., Danvers, MA, USA), and fluorescence *in situ* hybridization (FISH) for ALK.

## Materials and methods

### Materials and study design

**Figure 1:**
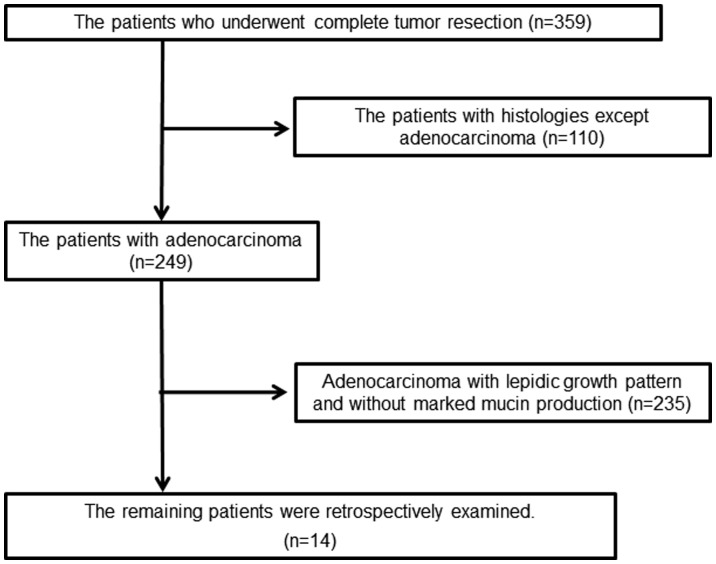
Study design. A total of 359 patients with primary lung carcinoma were enrolled. Out of the 359 patients, 110 cases with non-adenocarcinoma histologies were excluded. Out of the remaining 249 cases, 14 were selected based on mucin production.

The present retrospective study examined 359 patients with primary lung carcinoma whose tumors had been completely surgically removed at the Department of Surgery, Kurume University (Kurume, Fukuoka, Japan), between 2002 and 2011. Out of the 359 patients, 110 patients who were not histologically diagnosed with adenocarcinoma were excluded. The remaining 249 patients were histologically diagnosed with adenocarcinoma. Out of the 249 cases, 14 cases were selected due to the presence of marked mucin production (Fig. 1). The present study was approved by the ethical committee of Kurume University (no. 104). Written informed consent was obtained from the paitents.

### Immunohistochemistry

IHC for ALK was performed on paraffin-embedded sections by two different procedures. Two antibody preparations specific for the intracellular region of ALK were used, namely 5A4 (Nichirei Biosciences, Inc.) and D5F3 (Cell Signaling Technology, Inc.). The paraffin-embedded tissue samples were cut to a 4-μm thickness, examined on a coated slide glass and labeled with the antibodies as aforementioned. IHC using clone 5A4 was performed with the ALK detection kit, according to the manufacturer’s instructions (Nichirei Biosciences, Inc.). This kit applies an iAEP method (15). IHC with clone D5F3 (rabbit monoclonal antibody; 1:200) was performed on the fully automated Bond-Max system (Leica Biosystems Newcastle, Ltd.) using onboard heat-induced antigen retrieval with ER2 for 20 min and a refine polymer detection system (Leica Biosystems Newcastle, Ltd.). The histological specimens were incubated with the primary antibody for 14 min at room temperature and DAB was used as the chromogen in all IHC experiments.

The immunoreactive distribution was graded into five levels according to the distribution of immunoreactive tumor cells: 0, when there were no positive cells; 1+, when the area covered by immunoreactive cells was 1–25%; 2+, when the area was 26–50%; 3+, when the area was 51–75%; and 4+, when the area was >76%. The staining intensity for ALK was graded into four levels following the procedure of a previous study (16): 0, no staining; 1+, faint cytoplasmic staining; 2+, moderate, smooth cytoplasmic staining; and 3+, intense granular cytoplasmic staining. The total score was obtained from the immunoreactive distribution multiplied by the staining intensity score.

### FISH for ALK rearrangement

To identify ALK rearrangements, FISH was performed on formalin-fixed, paraffin embedded tumors using a break-apart probe for ALK (Vysis LSI ALK Dual Color Probe; Abbott Molecular, Des Plaines, IL, USA). FISH for ALK locus rearrangement was considered positive if ≥14% of the tumor cells counted exhibited a split signal. The criteria for probe signal interpretation in ≥100 interphase nuclei were as follows: i) Separated green and orange signals or single red signals identified the cells with rearranged ALK; and ii) overlapping of red and green signals (yellowish) indicated the cells in which ALK was not rearranged.

### Status of the EGFR tyrosine kinase domain

Genomic DNA was extracted from paraffin-embedded tissues using a QIAamp DNA Micro kit (Qiagen Inc., Valencia, CA, USA). Polymerase chain reaction (PCR) was performed using the TaqMan Mutation Detection Assay (Applied Biosystems Life Technologies, Carlsbad, CA, USA) using StepOneTM Real Time PCR System and Mutation DetectorTM Software version 1.0 (Applied Biosystems Life Technologies), according to the manufacturer’s instructions. To identify the EGFR mutation, the following primers were used: Hs00000228_mu, Hs00000157_mu, and Hs00000102_mu. The PCR solution (Applied Biosystems Life Technologies) consisted of 10 μl TaqMan® Genotyping Master Mix, 2 μl genomic DNA, 6 μl nuclease-free water and 2 μl TaqMan Mutation Detection Assay. The PCR conditions were as follows: One cycle at 95°C for 10 min, five cycles at 92°C for 15 sec and 1 min at 58°C, 40 cycles at 92°C for 15 sec and 1 min at 60°C.

### Statistical analysis

The association between cases with and without ALK rearrangement was examined by Student t-test or χ^2^ test. P<0.05 was considered to indicate a statistically significant difference*.*

## Results

**Figure 2:**
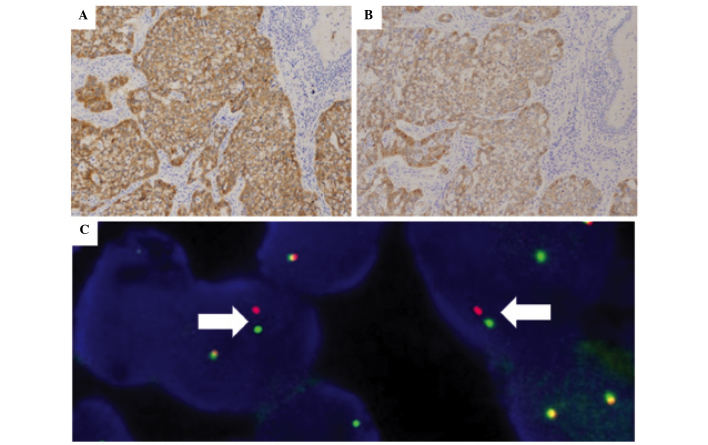
Representative images of immunohistochemical staining for ALK using two different methods and fluorescent *in situ* hybridization. (A) Clone 5A4 with iAEP and (B) clone D5F3 with the Bond-Max system (x200 magnification). (C) The break-apart probe for ALK shows a split signal, indicated by white arrows.

**Figure 3:**
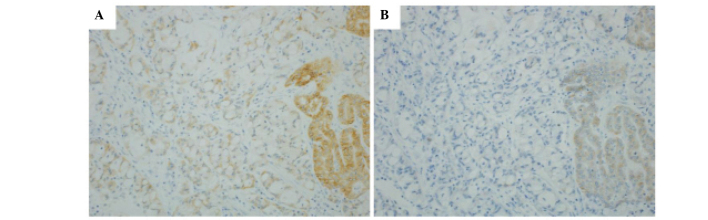
Immunohistochemical staining of a solid signet ring cell component with (A) anti-ALK antibody clone 5A4 with iAEP and (B) clone D5F3 with the Bond-Max system (x200 magnification). The solid signet ring cell component shows a weaker staining intensity in the two antibodies.

Eight cases of ALK-positive lung carcinoma were found by IHC. FISH revealed that seven out of eight (87.5%) cases possessed ALK rearrangement (Fig. 2A–C). The clinicopathological findings are shown in Table I. The IHC scores of the two different antibodies almost correlated with each other (Table II), but there was no statistical difference. The ALK-positive area was widely distributed in each method. The distribution score was 4 (>76%) or >3 (>51%) in both methods. However, tumor components exhibiting a solid signet ring cell pattern demonstrated a weaker cytoplasmic signal in D5F3 with the Bond-Max system in four cases (Fig. 3A and B).

In order to screen effectively, the cases with adenocarcinoma histology with mucin production were focused on and 14 cases were selected. Out of the 14 cases, seven cases were identified as ALK-positive lung carcinoma. All cases demonstrated the previously described characteristic histological patterns, such as a mucinous cribriform and/or solid signet ring cell pattern (Table I). One case, which had characteristic histological patterns of ALK-positive lung carcinoma, was identified by IHC as possessing ALK expression, but FISH demonstrated that the carcinoma lacked ALK rearrangement. This case exhibited neither split signals for ALK nor normal signals. ALK-positive lung carcinoma was significantly predominant for male patients in this study. However, these findings may be non-specific for ALK-positive lung carcinoma due to the small sample size.

EGFR mutation was not found in any of the seven ALK-positive lung carcinomas.

## Discussion

An ideal method for determining the presence of ALK-rearrangement has yet to be established. However, according to the Food and Drug Administration, it is necessary to confirm ALK-rearrangement by FISH in order to use the ALK inhibitor, crizotinib (17). Although FISH analysis is essential for the clinical usage of crizotinib in the United States, a previous study has demonstrated that initial screening by FISH alone does not detect all cases with ALK-positive lung carcinoma (8). In addition, the interpretation of FISH for ALK in NSCLC tends to be difficult, as ALK-positive lung carcinoma possesses an intrachromosomal rearrangement, resulting in a relatively close separation of the break-apart probes (16). Discordances between IHC and FISH have been thoroughly investigated in HER2/neu-positive breast carcinoma. The discordances between IHC and FISH are reported to be in the range of 10–20% (18–20). This may result from delayed or prolonged fixation, errors in IHC interpretation, HER2/neu antibody reagent limitations and the different antibodies used (20), a lack of interlaboratory standardization and reproducibility in the interpretation of the results (21) or genetic heterogeneity, which can contribute to positive IHC and negative FISH tests (22,23). At present, as there is no definitive recommendation from the laboratories performing IHC and FISH for ALK rearrangement in NSCLC, it is necessary to develop simple and accurate screening systems. Therefore, the present study focused on IHC for ALK rearrangement using two different antibodies and procedures. Previous studies have reported that IHC is a reliable screening tool for ALK-positive lung carcinoma (15,24–26). In the present study, it was demonstrated that the IHC score for ALK rearrangement using rabbit monoclonal antibody D5F3 with the Bond-Max system was similar to that of antibody 5A4 with the iAEP method. The combination of the D5F3 antibody and the Bond-Max system is simple and much cheaper than the iAEP method. This combination could also be suitable for the screening of ALK-positive lung cancer. Additionally, the D5F3 antibody could detect numerous variants of EML4-ALK or an unknown oncogenic fusion (27). In the present study, as the distribution scores of ALK in each method were relatively high, IHC for ALK may have low heterogeneity, suggesting that using IHC for ALK could be useful in limited tissue samples, such as in biopsy specimens or cytology, for the screening of ALK-positive lung carcinoma (28). Recently, Takamochi *et al* also described the expression of ALK on IHC as homogeneous (29). By contrast, Selinger *et al* reported that tissue microarray samples from the same tumor demonstrated heterogeneity of IHC for ALK when exhibiting weak or faint staining (30). Although explanations for these discrepancies remain elusive, the different samples and IHC procedures utilized in each study may be associated. In the present study, tumor components exhibiting a solid signet ring cell pattern demonstrated a slightly weak cytoplasmic signal, which may be attributed to abundant cytoplasmic mucin. As this component is known to be one of the characteristic histological findings in ALK-positive lung carcinoma, an awareness of marked mucin production is necessary to avoid an underestimation of the proportion of ALK-rearranged cells. Therefore, the assessment of IHC for ALK in limited tissue samples should be performed with care, particularly when the IHC signal is weak in a solid signet ring cell component.

Among the eight cases in the present study that were confirmed to exhibit ALK expression by IHC, seven cases were demonstrated to possess ALK rearrangement by FISH. The sensitivity of FISH for ALK was 87.5%. This sensitivity was lower than that of previous studies. The one case in which FISH did not confirm ALK rearrangement possessed high IHC scores for ALK expression and demonstrated characteristic histological patterns. A few studies have documented that all cases demonstrating a strong intensity of ALK on IHC were also revealed to have ALK rearrangement by FISH (16,28). The precise reasons for the discrepancy observed in the present study remain elusive. However, the case that lacked ALK rearrangement according to FISH was >10 years old. Neither a split signal for ALK nor a normal signal could be detected in this case. This may have resulted from degeneration of the DNA or from delayed or prolonged fixation. Thus, the ALK test should be performed promptly in accordance with the College of American Pathologists, International Association for the Study of Lung Cancer and Association for Molecular Pathology (CAP/IASLC/AMP) guidelines (31).

Although 14 cases were enrolled in the present study on the basis of the presence of characteristic histological patterns, any case with adenocarcinoma should not be excluded from the possibility of ALK-positive lung carcinoma without IHC or FISH for ALK rearrangement, in accordance with the CAP/IASLC/AMP guidelines (31). None of the ALK-positive lung carcinomas harbored coexisting EGFR mutations in the present study. These findings are consistent with those of previous studies, demonstrating that ALK positive lung carcinoma is exclusive of EGFR mutations (8,11,13).

In conclusion, a combination of the methodologies of IHC and FISH could be suitable for screening for ALK-positive lung carcinoma. The IHC for ALK, using the rabbit monoclonal antibody D5F3 and the Bond-Max system, demonstrated similar results to those of the iAEP method and showed low heterogeneity. As the present study is on a small scale, further expanded studies using larger cohorts should be conducted in order to confirm the validity of screening for AKL-positive lung carcinoma using the D5F3 antibody.

## Figures and Tables

**Table I:** Clinicopathological features of adenocarcinoma with and without ALK rearrangement.

Feature	ALK^+^ (n=7)	ALK^−^ (n=7)	P-value
Age, years (±SD)	59.4±8.9	59.3±12.0	0.98
Gender (M:F), n	4:3	0:7	0.018
Median smoking habit, BI	0	0	1
Histomorphology, %			
Any papillary pattern	28.6	43.9	0.53
Any acinar pattern	85.7	100.0	0.30
Mucinous cribriform^a^	57.1	0.0	0.018
Any solid pattern	85.7	87.5	1
Solid signet ring cell^a^	85.7	14.3	0.0075

a Parameters with a statistically significant difference according to the χ^2^ test.

BI, Brinkman index; ALK, anaplastic lymphoma kinase; M, male; F, female; NS, no significant difference; SD, standard deviation.

**Table II:** Immunohistochemical stain score of the two antibodies and procedures.

Case	ALK 5A4 with iAEP^a^	ALK D5F3 with Bond-Max system^a^
1	12 (3×4)	12 (3×4)
2	12 (3×4)	12 (3×4)
3	8 (2×4)	6 (2×3)
4	8 (2×4)	8 (2×4)
5	12 (3×4)	12 (3×4)
6	8 (2×4)	6 (2×3)
7	9 (3×3)	8 (2×4)
Average	9.9	9.1

a Immunohistochemical staining score was obtained from the immunoreactive distribution multiplied by the staining intensity score (in parentheses).

ALK, anaplastic lymphoma kinase; iAEP, intercalated antibody-enhanced polymer.
